# Real-World Accuracy of a Continuous Glucose Monitoring System after Radiologic Exposure

**DOI:** 10.1155/2024/2210509

**Published:** 2024-08-07

**Authors:** Siobhan Tellez, Lindsey Hornung, Emily Smith, Andrew Trout, Samuel Brady, Colleen Lowe, Joshua Courter, Maisam Abu-El-Haija, Deborah Elder

**Affiliations:** ^1^ Division of Endocrinology Cincinnati Children's Hospital Medical Center, Cincinnati, Ohio, USA; ^2^ Division of Biostatistics and Epidemiology Cincinnati Children's Hospital Medical Center, Cincinnati, Ohio, USA; ^3^ Department of Radiology Cincinnati Children's Hospital Medical Center, Cincinnati, Ohio, USA; ^4^ Department of Radiology University of Cincinnati College of Medicine, Cincinnati, Ohio, USA; ^5^ Department of Pediatrics University of Cincinnati College of Medicine, Cincinnati, Ohio, USA; ^6^ Division of Pediatric General and Thoracic Surgery Cincinnati Children's Hospital Medical Center, Cincinnati, Ohio, USA; ^7^ Division of Pharmacy Cincinnati Children's Hospital Medical Center, Cincinnati, Ohio, USA; ^8^ Division of Gastroenterology Hepatology and Nutrition Cincinnati Children's Hospital Medical Center, Cincinnati, Ohio, USA

## Abstract

**Background:**

The increasing use of continuous glucose monitor (CGM) necessitates a review of variables that impact accuracy and interrupt use. Manufacturer recommendations include removing CGMs before diagnostic imaging, such as X-ray and computed tomography (CT). Early removal and replacement of CGM components present financial, clinical, and psychosocial burdens to the wearer and interrupt optimal management of diabetes for pediatric patients who receive a total pancreatectomy with islet autotransplantation (TPIAT). The study's aim was to evaluate the effect of scatter dose exposure during X-ray or CT if the CGM remained intact but outside the field of view (FoV).

**Materials and Methods:**

Participants were followed through the first 3 months after TPIAT surgery, managed diabetes with an insulin pump and CGM, and were routinely exposed to diagnostic imaging. Participants' CGMs were unshielded by a protective apron during any X-ray or CT procedures for the duration of the study period, and the transmitter was collected after expiration or removal. Glucometer data was collected from hospital records and home glucometer downloads. Mixed models were used to analyze absolute differences between matched CGM and glucometer values, and Clarke error grid analyses (EGA) were performed. Scatter dose exposure was derived using anthropomorphic phantoms and calculated retrospectively.

**Results:**

A total of 14 patients (median 12.2 years, 64% female) received a median of five diagnostic imaging procedures with a median cumulative scatter dose of 559 *µ*Gy. The absolute difference between the CGM and glucometer values was not significantly associated with the cumulative scatter dose (*p*=0.17) or time from TPIAT (*p*=0.24) when analyzed in a mixed model. Regardless of scatter dose exposure, time from TPIAT, or glucometer, ≥98% of glucose values fell within zones A and B on EGA.

**Conclusion:**

Scatter dose exposure from diagnostic imaging did not affect the clinical accuracy of CGM values for the duration of transmitter use. Leaving CGM components in place when not in the FoV during diagnostic imaging successfully mitigated interruptions to use and undue burden or cost to participants.

## 1. Introduction

Continuous glucose monitors (CGM) are disposable or implantable devices that provide interstitial glucose readings at a frequency of up to every minute [1]. The volume of information provided by CGM systems, compared with standard self-monitoring with blood glucose meters, improves the wearer's ability to optimize glycemic management while simultaneously reducing the risk of severe hypoglycemia and diabetic ketoacidosis (DKA) [2, 3, 4]. The benefits of real-time CGM use on glycemic outcomes are well documented, such as lowering HbA1C, reducing the frequency of severe hypoglycemic events and reducing DKA hospitalizations [1, 2, 4, 5]. The American Diabetes Association (ADA) recommends the use of CGM systems for individuals on insulin therapy, regardless of diabetes type [1]. Advancement in CGM technology has led to the availability of factory calibrated CGM systems. These devices are approved to replace traditional glucometer testing for diabetes management and no longer require intermittent calibration from glucometer readings to maintain accuracy. However, individuals using CGM systems are still recommended to have access to alternative methods of blood glucose monitoring, as there are situations that necessitate validation of the interstitial reading or complete removal of the device. Such situations include exposure to interfering substances [6, 7, 8], settings where glucose levels change rapidly (>2 mg/dL/min) [1], and removal for diagnostic imaging such as digital radiography (DR), computed tomography (CT), or magnetic resonance imaging (MRI) [9]. Given the increasing use of CGM readings (almost exclusively) for diabetes management decisions, both wearers and healthcare professionals need information on the accuracy of these devices and of the variables that impact accuracy.

Children and adolescents with debilitating chronic or acute recurrent pancreatitis who receive a total pancreatectomy with islet autotransplantation (TPIAT) are immediately started on an intensive insulin regimen to protect and promote islet engraftment. At our center, a Dexcom G6 CGM is provided to all TPIAT recipients after surgery, augmenting point-of-care glucometer testing to reach a target glycemic range of 80–120 mg/dL [10]. This early CGM use has historically been interrupted repeatedly during the postsurgical period due to requisite diagnostic testing with DR, CT, and MRI. Devices are removed and replaced throughout the hospitalization, which increases the medical intervention burden on the patient, increases the cost burden to the family and care program, and increases medical equipment waste. Even after discharge, interruptions to CGM system use often continue as these patients may require repeat diagnostic imaging.

The Dexcom G6 CGM system (Dexcom, San Diego, CA) is labeled for wearers age 2 years and older [9] and is intended to replace fingerstick blood glucose testing as a standalone CGM system or in conjunction with automated insulin delivery systems [9]. The G6 system is comprised of three components: a receiver, a sensor, and a transmitter. The sensor is single-use, worn externally, and has a flexible filament that is inserted subcutaneously. The sensor filament collects interstitial glucose readings every 5 min [9]. All sensors are factory-calibrated for accuracy but can be calibrated with capillary glucose readings [9]. A reusable transmitter attaches to the sensor and transmits the readings to a receiver device or smartphone application along with an arrow system to indicate the current rate of rise or fall. The transmitter has circuitry for signal processing, uses Bluetooth® technology for connecting with devices and sharing data, and lasts for 90 days. Current manufacturer guidelines for this system are to avoid whole-body millimeter wave scanners (often used in airports), to avoid placing any G6 components through baggage X-ray machines, and to remove G6 completely for any MRI, CT, or diathermy [9]. Recommendations related to X-ray imaging for diagnostic purposes are not readily available, and the effect of X-ray exposure on the wearable components has not been extensively reported. An in-vitro study simulated X-ray exposure to sensor/transmitter dyads up to 80 gray (Gy) and studied glucose concentrations reported by the dyads over 21 days [11]. Of note, a direct exposure of 80 Gy represents an exposure 8,000 times or 100,000 times greater than a direct exposure from a typical patient's abdominopelvic CT or X-ray imaging examination, respectively. This study reported that the wearable components retained the basic functionality and data integrity and concluded that the G6 system was unlikely to be affected by diagnostic irradiation [11]. Although promising, this study did not study the entire lifespan of the transmitter, which is reusable for 90 days.

Due to the frequency of diagnostic imaging performed on pediatric TPIAT recipients that commonly results in interruption to CGM use, our aim was to determine the impact of radiation exposure on the clinically significant accuracy of Dexcom G6 and wearable components.

## 2. Materials and Methods

### 2.1. Study Design

The study was designed as a nonrandomized prospective trial with a target enrollment of 20 participants (IRB 2021-0831). CGM readings were assessed for accuracy and precision by comparing them to glucometer readings from the hospital point-of-care glucometer and a study-provided commercial glucometer until the Dexcom transmitter-initiated postsurgery was changed.

An ACCU-CHEK® Inform II glucometer (Roche Diagnostics Corporation, Indianapolis, IN, USA) was used during the hospitalization, and a study issued Contour® Next One glucometer (Ascensia Diabetes, Parsippany, NJ, USA) was used by participants after hospitalization. At the time of the study design, it was one of few home glucometers that provided an option for comma-separated value (CSV) file sharing with discrete values and precise time stamps. This permitted pairing with CGM values from Dexcom CSV file sharing. The use of a home glucometer permitted participants to use the glucometer for daily management and share data that was reflective of typical use. Information on accuracy for both meters was available from the manufacturer's website and showed similar accuracy when using capillary samples. When glucose values were <75 mg/dL, 99.3% (*n* = 135) of the ACCU-CHEK® glucometer values and 100% (*n* = 17) of the Contour® glucometer values were within ±15 mg/dL. When glucose values were ≥75 mg/dL, 99.9% (*n* = 1798) of the ACCU-CHEK® values and 99.7% (*n* = 355) of the Contour® values were within ±15 mg/dL. Time-stamped glucometer data and sensor values were collected from devices and compared for accuracy. The inclusion of both the hospital and home glucometers provided paired sample data following radiographic exposure for the duration of the transmitter.

The time to Dexcom G6 transmitter replacement, number, and type of imaging procedures involving diagnostic radiation exposure were collected. Hydroxyurea (HU)—frequently prescribed to TPIAT recipients as part of the postsplenectomy protocol—is known to influence glucose sensor values for 6–9 hr following administration [6, 7]. As a result, the daily administration time of this medication was also collected from hospital records and from family reports at outpatient appointments. Data 12 hr after HU administration that were potentially influenced by this interfering substance were excluded from the analysis. Data were collected at the following intervals: 1 week following discharge from the hospital (±3 days), 1 month after TPIAT surgery (±7 days), 2 months after surgery (±7 days), and 3 months after surgery (±7 days), or at the time of transmitter change. If the visit window for the 1-week postdischarge and 1-month postsurgery overlapped, they were combined into one interval.

### 2.2. Participants

Eligible participants for this study included patients who were scheduled to have a TPIAT at our academic pediatric medical center and use the Dexcom G6 CGM system following surgery. Exclusion criteria were the requirement of hemodialysis or peritoneal dialysis. From February 2022 through July 2023, 21 eligible participants were approached for inclusion, and 20 were enrolled. All participants were insulin-dependent immediately after surgery and through the first 3 months after surgery (standard care for all TPIAT recipients at our center). All participants were managed on intravenous insulin intraoperatively and while in the intensive care units, transitioned to an insulin pump on the endocrine unit, and continued insulin pump therapy after discharge [12]. All participants started the Dexcom G6 in the pediatric intensive care unit immediately following surgery, with sensor placement on the anterior thigh (standard practice for TPIAT recipients at our center) [10].

All participants provided parental or legal guardian permission (participants under 18 years old), assent (participants aged 11–17 years old), or informed consent signed by the patient (when 18 years old and older) for this study prior to their TPIAT surgery. A chart review was completed to collect demographic information, indication for TPIAT surgery, and date of admission, surgery, and discharge. Whether or not the participant was on hydroxyurea and the time it was taken was collected at each timepoint. Participation ended when patients attended the 3-month follow-up appointment postsurgery or when the Dexcom transmitter was changed (whichever came first).

### 2.3. Study Procedures

Participants were instructed to continue to wear the Dexcom sensor and transmitter (typically worn on the anterior thigh or upper arm post-TPIAT) if the devices were not in the radiologic field of view (FoV). The radiology staff were instructed not to manipulate the device and to leave the devices unshielded by a protective apron during DR and CT imaging. Parents or caregivers of the participants were similarly instructed that CGM components were to remain on the body and unshielded during DR and CT imaging.

During participants' post-TPIAT hospitalization, the hospital glucometer (HG) was used for glucose monitoring and medical decision-making as part of the standard of care [12]. The study team collected all glucometer readings from the electronic medical record after discharge to compare with the CGM readings. Participants were instructed to check their blood glucose on the study-issued glucometer (SIG) at least four times a day in the 10 days prior to each study interval. At each interval, all glucometer data in the past 90 days were collected for comparison with CGM readings. Participants continued to use the Dexcom G6 CGM to monitor glucose trends. They were educated on the use of the Dexcom G6 CGM post-TPIAT as part of comprehensive diabetes education during the hospitalization and were instructed to use glucometer readings (not CGM readings) for all insulin dosing decisions.

Ionizing radiation exposure (occurring from any diagnostic X-ray or CT scan during the study period) was assessed through chart review as well as family self-report at each interval. Data were collected on FoV of the imaging, indication for imaging, and date and time of imaging. Patients undergoing DR studies typically underwent chest, abdomen, or pelvis imaging examinations. Patients undergoing CT studies underwent chest or abdomen-pelvis imaging examinations. No patient had diagnostic imaging of the thighs or upper arms (sites where the sensor/transmitter is worn). Therefore, when estimating the radiation exposure to the G6 wearable components, no components were assumed to be directly irradiated in the DR or CT scan FoV. Instead, components were assumed to have been exposed to secondary, “scatter” radiation that spreads in different directions when the X-ray beam interacts with the patient being imaged. The quantity of scatter incident upon the wearable components was affected by two principles: the volume of tissue exposed by x-rays and the distance the components were from the exposed tissue FoV. Adult-sized patients will produce more scatter than pediatric-sized patients, but they will also have proportionately longer arms and legs so that the potential increase in scatter from adult-sized patients will be offset by the wearable components being further away from the imaging FoV; conversely, pediatric patients will produce less scatter-by-volume even though they have shorter arms and legs, and closer wearable components to the imaging FoV. To create a scatter field model for this study, an anthropomorphic phantom representing an average-sized adult, made from tissue equivalent polymers (ATOM, CIRS, Norfolk VA) and a cylindrical shaped phantom (32 cm in diameter), made from acrylic, were imaged in similar configurations and with similar X-ray technique factors as the patients in this study. The scattered radiation from the anthropomorphic phantoms was measured with a 180-cc ionization chamber (10x180, Radcal, Monrovia, CA) at different distances from the anthropomorphic phantoms for both DR and CT imaging geometries.

The measured scattered dose data were fit using an exponential regression curve. Separate scatter data were derived for different examination acquisition factors such as tube potential (i.e., kV) and beam quality (i.e., half value layer). The scatter data were further scaled by the unique tube current-time product (i.e., mAs) used to acquire the individual patient examinations and the different distances from the examination FoV boundaries to the G6 wearable components. The scattered dose to the wearable components was calculated for each patient examination, assuming that the components were either located in the upper thigh or upper arm of each patient. Since the dose calculations for this study were retrospectively acquired, no physical measurement of the location of the components for each patient was available; thus, for each patient, the components in the upper thigh were calculated to be located as the halfway distance between the patient's femoral greater trochanter to their lateral condyle, and the components in the upper arm were calculated as the halfway point between the head of the humerus to the lateral epicondyle; the arms were assumed to be over the head of the patient for all examination types, which is the standard positioning at our institution.

### 2.4. Statistical Analysis

Data were analyzed using SAS®, version 9.4 (SAS Institute, Cary, NC). Continuous data were summarized as means with standard deviations or medians with interquartile ranges (IQR: 25th−75th percentiles), while categorical data were summarized as frequency counts and percentages. CGM results were matched to the glucometer reading with the closest collection time (within 5 min of the CGM result time). Clarke error grid analyses (EGA) were performed to compare CGM values to glucometer glucose values. A *p*-value <0.05 was considered statistically significant. Generalized linear mixed models with random effects to account for repeated measures by the same subject over time were used to assess whether the difference or absolute difference between the CGM and glucometer values was affected by time and/or cumulative scatter dose. Hydroxyurea was typically administered between 9:00 am and 11:00 am during the hospitalization, and families continued this schedule after discharge. To avoid the effects of hydroxyurea on the CGM sensor, we only used time-matched CGM and glucometer data points between 9:00 pm and 8:59 am due to not knowing precisely when the patient was given hydroxyurea after discharge.

## 3. Results

Twenty patients were consented for study participation. Two participants were withdrawn before any data collection occurred due to canceled surgeries, two participants were withdrawn when the Dexcom transmitter was removed immediately postsurgery (one participant was not able to tolerate wearing the device, and one participant misunderstood instructions to change the sensor, and disposed both the sensor and the transmitter), which prevented data collection, and two did not have radiation exposure during the time interval while wearing the device (excluded during data analysis). A total of 14 patients completed the study with a median duration of follow-up of 2.8 months (Table 1). The median age at the time of TPIAT was 12.2 years (IQR: 9.4–15.1 years), 64% of the participants were female, and 86% identified as white (Table 1). Participants underwent a median of 5 (IQR: 3–6) imaging procedures with cumulative total scatter dose exposure of 559 *µ*Gy (IQR: 59–1211 *µ*Gy) (Table 1). Most participants had private insurance coverage (64%) but 43% had Medicaid insurance as either primary or supplementary coverage (one patient had both private and Medicaid). No CGM components were removed from any participants' radiologic procedures for the duration of the study.

**Figure 1:**
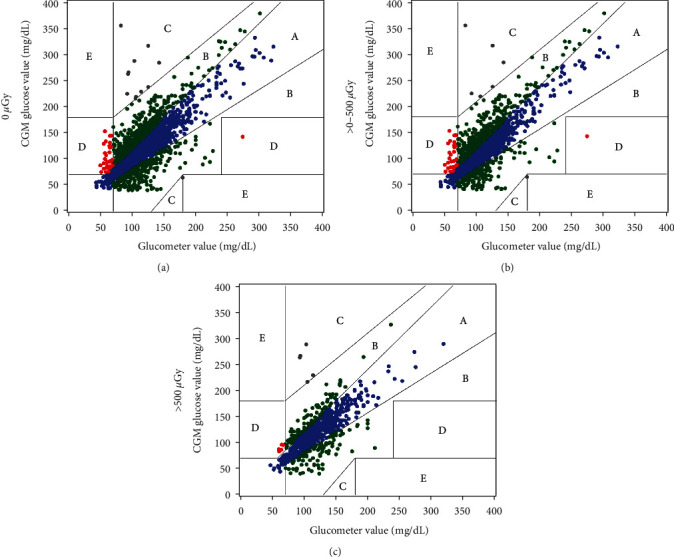
Clarke error grid analysis grouped by cumulative scatter dose. (a) EGA plot for 0 *µ*Gy, (b) EGA plot >0–500 *µ*Gy, and (c) EGA plot for >500 *µ*Gy.

In total, there were 4168 CGM-glucometer matched data points analyzed, 67% of which were collected from the HG (ACCU-CHEK®). A total of 1375 matched data points were collected from the participants in the outpatient setting using the SIG (Contour®). There were 1435 matched pairs before radiation exposure and 2,733 matched data points after radiation exposure. To not only assess preradiation compared to postradiation exposure but also the magnitude of exposure, we split postradiation exposure into >0−500 *µ*Gy and >500 *µ*Gy to make sure the higher ends of exposure did not impact the device. Based on EGA comparisons, pre-exposure pairs (0 *µ*Gy) were compared to those with cumulative scatter doses >0−500 *µ*Gy and >500 *µ*Gy, which had similar proportions in Zones A and B (98.5% versus 97.9% versus 98.9%, respectively, *p* = 0.16) (Table 2 and Figure 1). When grouped by time from TPIAT, those proportions were not different, with 98.3% within Zones A and B at 0–1 months postoperatively, 98.0% at 1–2 months, and 99.7% at 2+ months (*p* = 0.13) (Table 2). Matched data points were grouped by meter (HG versus SIG) for comparison. There was no difference in the proportion of data points that fell within Zones A and B (98.4% versus 98.1%, respectively, *p* = 0.46) (Table 2). Among the data points that used the SIG, the majority fell in Zone A compared to Zone B (74.2% versus 23.9%). There was no difference in the proportion of data points in Zone D (indicative of a potentially dangerous failure to identify hypo- or hyperglycemia) based on the cumulative scatter dose exposure or between meters; all groups had <2% (Table 2).

**Figure 2:**
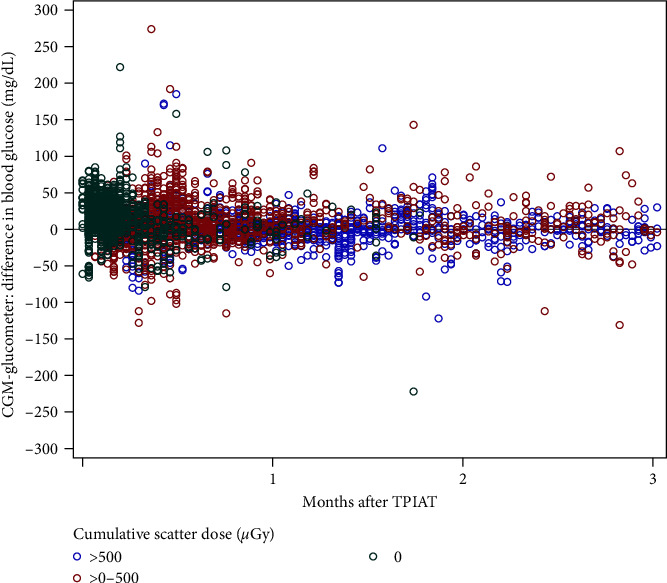
Difference in CGM-glucometer matched readings over time grouped by cumulative scatter dose exposure.

When analyzed using a mixed model, the absolute difference between the CGM and glucometer values was not significantly associated with the cumulative scatter dose (*p* = 0.17) or time from TPIAT (*p* = 0.24). The difference between CGM-glucometer-matched values, grouped by cumulative scatter dose, is shown over time in Figure 2.

## 4. Discussion

The data presented in this study are similar to previously reported findings that the accuracy of the Dexcom G6 system is not significantly impacted by indirect scatter dose radiation exposure, but expand on those findings by encompassing the life of the transmitter (the component with the longest continuous use) [11]. EGA of cumulative DR and CT exposure showed no difference in the proportions of clinically acceptable readings over time and nearly all values within Zones A and B at all intervals. There was no significant difference in EGA for the proportions in Zones A and B for preradiation exposure compared to postradiation exposure. There was also no difference in the proportions of clinically acceptable readings between the two glucometers used in this study, which suggests the use of a commercial glucometer, rather than a reference instrument, did not confound results. We found that more data points from the SIG pairs fell within Zone A compared to the HG pairs (74.2% versus 61.4%), meaning the CGM values were within 20% of the commercial glucometer with “typical use” by participants.

All study participants were able to keep the CGM components in place during all DR and CT imaging. If CGM components had been removed for all imaging procedures, the study participants would have averaged five removals and reinsertions. Some participants would have had up to nine removals and reinsertions. Not removing the CGM components reduced the need for reinsertions by 100%, which eliminated a potential source of physical and/or psychosocial stress for patients and caregivers. It also prevented the wasting of sensor and transmitter supplies, which addressed caregiver concerns of cost. True out-of-pocket costs cannot be reported due to differences in insurance coverage and the availability of manufacturer pharmacy savings benefits or assistance programs (including replacement of eligible CGM components from Dexcom customer support). However, based on publicly available average wholesale pricing, study participants could have encountered out-of-pocket costs of about $750 on average (nearly $1,400 for those who received nine imaging procedures) within the first 3 months of CGM use.

Most TPIAT patients and families travel from out of state to receive care at our center. Our program routinely encounters challenges with the coverage and provision of diabetes devices and supplies due to variable policies and restrictions with private, but more so with Medicaid plans (unpublished data). Nearly half (43%) of the study participants were enrolled in Medicaid insurance plans at the time of study participation. While challenges also exist for those with private insurance coverage, the consequences of the more restrictive Medicaid plans can be harder to mitigate. For example, many Medicaid plans require the prescribing provider and dispensing pharmacies to be located within the state issuing the plan. Reducing the depletion of diabetes equipment after TPIAT alleviates the burden for key stakeholders: the families who navigate pharmacy and insurance roadblocks, the clinical staff who process prior authorizations and denials of coverage, and the prescribing providers who appeal for coverage.

For children and adolescents who undergo TPIAT at our center, the early initiation of the Dexcom G6 CGM has been a key component of maintaining optimal glycemic management immediately after TPIAT [12, 13]. However, there are very few publications on the outpatient use of CGMs in this population, particularly related to the patients' experience. The optimization of patient experience in the use of diabetes technology is beneficial as the majority of TPIAT patients have long-term insulin requirements. A high-volume center for pediatric TPIAT studied the acceptability, accuracy, and feasibility of CGM use compared with a glucometer in the immediate postoperative period [14]. They found overall patient satisfaction with CGM use to be high, and that most participants would recommend its use to other individuals with diabetes [14]. Although qualitative data on the participant's experience with CGM fell outside the scope of our study, the aim was a direct response to the feedback we received from families and caregivers of our TPIAT patients on how frequent removal and reinsertion of the Dexcom impacted their care and their experience of the CGM. Our hope is that the results of this study help optimize our patients' experience with the Dexcom G6 CGM beyond the initial postoperative period and contribute to a better understanding of the unique variables that influence CGM performance in these patients.

This study is limited by the single-center design. The pairing of CGM values with glucometer values—rather than venous blood glucose value—limits the interpretation of these results due to the inherent variability across glucometers and the lack of standardization in glucose sampling. However, the use of glucometer-CGM matched pairs yielded enough data points for analysis and leveraged the existing standard of care for self-monitoring of blood glucose. Additionally, the glucometer-CGM pairing reflects the user experience and the typical data available for clinical interpretation and, therefore, is more applicable to the aim of the investigation. There was a higher proportion of HG-matched data points compared with commercial glucometer-matched data points, which limits the generalizability of these results. This was due to an overall decrease in the frequency of finger sticks by caregivers once discharged from the hospital. The quantity of matched glucometer-CGM data points available for analysis was further limited due to hydroxyurea use. Data collection parameters were restricted to pairs that fell outside the window of influence from hydroxyurea to minimize the potential impact on EGA and modeling the absolute difference [6]. However, this could not completely account for any individual variation in medication metabolism. Variables such as user error, individual biochemical interaction with the sensor filament, and the increased CGM variability on the first day of each sensor could not be accounted for in the data analysis. Population-specific variables—such as medication interference, the strict glycemic targets, limited glycemic variability, and fluid shifts during postoperative recovery—affect the interpretation of CGM accuracy and performance, which limits the application of these findings to individuals with other types of diabetes. All study participants had postsurgical abdominal incisions, which restricted the placement of the sensor to the extremities for the duration of the study period. Further studies are needed to evaluate more direct exposure (i.e., sensors within the FoV) and to compare with newer CGM systems that have integrated the transmitter into the sensor, thus reducing the likelihood of cumulative exposure over the lifetime of the sensor.

## 5. Conclusion

Pediatric patients using Dexcom G6 CGMs after total pancreatectomy did not remove the CGM wearable components during DR and CT imaging, saving an average of 5 removals and reinsertions per patient. We found that nearly 100% of CGM values (paired with glucometer values) were clinically accurate, and over 60% of those values were within 20% of the reference glucometer for the duration of the transmitter and regardless of the cumulative scatter dose exposure. The absolute difference between CGM and glucometer values was also not significantly associated with scatter dose radiation exposure over the lifespan of the transmitter. We conclude that the scatter dose exposure from diagnostic imaging experienced by participants did not impact Dexcom device accuracy, and these device components may be left on the body, unshielded, during diagnostic imaging such as DR and CT if they do not obstruct the FoV.

## Figures and Tables

**Table 1:** Patient characteristics.

	TPIAT *n* = 14
Age at TPIAT (years)	12.2 (9.4–15.1) (8.0–19.8)
Sex	
Female	9 (64%)
Male	5 (36%)
Race	
White/Caucasian	12 (86%)
Other	2 (14%)
Ethnicity (non-Hispanic/Latino)	10/13 (77%)
BMI percentile	79.4 (46.1–98.2)
Insurance	
Private	9 (64%)
Self	0 (0%)
Medicaid	6 (43%)
Other	0 (0%)
Total follow-up time (months)	2.8 (1.9–2.9) (0.5–3.0)
Cumulative scatter dose (*µ*Gy)	559 (59–1,211) (18–1,811)
Number of procedures (DR and CT)	5 (3–6) (1–9) 5 ± 2

Data presented as median (25th–75th percentile) (min–max) or mean ± SD or *n* (%).

**Table 2:** Clarke error grid analysis (EGA).

EGA groupings	Zones A and B (%)	Zone A (%)	Zone B (%)	Zone C (%)	Zone D (%)
CGM-HG ^*∗*^	98.4	61.4	37.0	0.5	1.1
CGM-SIG^†^	98.1	74.2	23.9	0.2	1.7
Months from TPIAT					
0–1	98.3	62.7	35.6	0.5	1.3
1–2	98.0	76.1	21.8	0.3	1.7
2+	99.7	78.5	21.2	0.0	0.4
Cumulative scatter dose					
0 *µ*Gy	98.5	56.2	42.2	0.4	1.2
>0–500 *µ*Gy	97.9	66.9	31.1	0.4	1.7
>500 *µ*Gy	98.9	78.1	20.8	0.55	0.55

^ ^*∗*^^HG, hospital glucometer; ^†^SIG, study issued glucometer.

## Data Availability

Data will be available upon request from the authors.
